# Mechanisms of ubiquitin-independent proteasomal degradation and their roles in age-related neurodegenerative disease

**DOI:** 10.3389/fcell.2024.1531797

**Published:** 2025-02-07

**Authors:** Taylor R. Church, Seth S. Margolis

**Affiliations:** ^1^ Department of Biological Chemistry, The Johns Hopkins University School of Medicine, Baltimore, MD, United States; ^2^ Solomon H. Snyder Department of Neuroscience, The Johns Hopkins University School of Medicine, Baltimore, MD, United States

**Keywords:** neurodegenerative disease, ubiquitin independent, protein degradation, proteasome, Alzheimer's disease, Huntington's disease, Parkinson's disease, oxidative stress

## Abstract

Neurodegenerative diseases are characterized by the progressive breakdown of neuronal structure and function and the pathological accumulation of misfolded protein aggregates and toxic protein oligomers. A major contributor to the deterioration of neuronal physiology is the disruption of protein catabolic pathways mediated by the proteasome, a large protease complex responsible for most cellular protein degradation. Previously, it was believed that proteolysis by the proteasome required tagging of protein targets with polyubiquitin chains, a pathway called the ubiquitin-proteasome system (UPS). Because of this, most research on proteasomal roles in neurodegeneration has historically focused on the UPS. However, additional ubiquitin-independent pathways and their importance in neurodegeneration are increasingly recognized. In this review, we discuss the range of ubiquitin-independent proteasome pathways, focusing on substrate identification and targeting, regulatory molecules and adaptors, proteasome activators and alternative caps, and diverse proteasome complexes including the 20S proteasome, the neuronal membrane proteasome, the immunoproteasome, extracellular proteasomes, and hybrid proteasomes. These pathways are further discussed in the context of aging, oxidative stress, protein aggregation, and age-associated neurodegenerative diseases, with a special focus on Alzheimer’s Disease, Huntington’s Disease, and Parkinson’s Disease. A mechanistic understanding of ubiquitin-independent proteasome function and regulation in neurodegeneration is critical for the development of therapies to treat these devastating conditions. This review summarizes the current state of ubiquitin-independent proteasome research in neurodegeneration.

## Introduction

Neurodegeneration causes an irreversible decline in cognition and motor coordination due to the progressive breakdown of neuronal structure and function. A defining feature of neurodegeneration is the accumulation of misfolded protein aggregates, which are toxic to the cell and cause neuronal damage by disrupting essential cellular processes. A fundamental mechanism in the formation of these aggregates is disruption of neuronal proteostasis, the balance of protein synthesis and degradation. This is mediated in part through the proteasome, a multi-subunit protease complex responsible for the majority of protein degradation, including the misfolded and damaged proteins implicated in neurodegenerative diseases (Zheng et al., 2016; Türker et al., 2021; Cuanalo-Contreras et al., 2023; Davidson and Pickering, 2023). The proteasome functions through multiple proteolytic mechanisms based on its composition and interactors.

The cell’s main degradative machinery, called the 26S proteasome, consists of a cylindrical 20S core particle (20S) that contains catalytic sites for proteolysis and a 19S regulatory cap (19S) that acts as a proteasome activator (PA) to facilitate recognition, unfolding, and rapid degradation of substrates. The 26S proteasome is the central hub for the ubiquitin-proteasome system (UPS), a catabolic pathway that targets proteins for destruction through 1) covalent attachment of polyubiquitin chains by a series of ubiquitin ligases, 2) recognition and de-ubiquitination by the 19S cap, and 3) ATPase-dependent unfolding and translocation of substrate proteins to the interior of the 20S core for degradation (Hershko et al., 1981; Hershko and Ciechanover, 1992; Ciechanover and Schwartz, 1998). The catalytic subunits of the 20S core include β5 (PSMB5; chymotrypsin-like activity), β2 (PSMB7; trypsin-like activity), and β1 (PSMB6; caspase-like activity), which cleave peptide bonds with different specificities and are responsible for the breakdown of proteins into short peptides (Baumeister et al., 1997; Kisselev et al., 1999; Unno et al., 2002). These peptide products are then used as a source of amino acids for biosynthesis or for other cell type-specific functions including antigen recognition, modulation of neuronal signaling, and intercellular communication (Vabulas and Hartl, 2005; Basler et al., 2013; Ramachandran and Margolis, 2017; Limanaqi et al., 2019; Türker et al., 2024). While the UPS is the best-characterized mechanism of proteasome activity (Bingol and Schuman, 2005; Patrick, 2006; Yi and Ehlers, 2007) and extensive reviews have been written on its role in neurodegenerative diseases (Ciechanover and Brundin, 2003; Dantuma and Bott, 2014; Zheng et al., 2016; Watanabe et al., 2020; Schmidt et al., 2021; Davidson and Pickering, 2023), many questions remain which are being actively explored and which will not be the focus of this review.

In addition to the UPS, the 20S proteasome is highly abundant and found with a broad set of activators and associated proteins important for various proteolytic functions, especially those affected by neurodegenerative disease (Fabre et al., 2014; Opoku-Nsiah and Gestwicki, 2018; Türker et al., 2023). While 20S proteasomes were previously believed to be non-functional without a regulatory 19S cap, increasing evidence has indicated unique, ubiquitin-independent roles of the 20S core particle and its interacting partners, particularly in degradation of intrinsically disordered, oxidized, or misfolded proteins (Jariel-Encontre et al., 2008; Baugh et al., 2009; Ben-Nissan and Sharon, 2014; Erales and Coffino, 2014; Opoku-Nsiah and Gestwicki, 2018; Davidson and Pickering, 2023), important hallmarks of neurodegeneration. Because a large portion of proteins in the human genome contain intrinsically disordered regions under physiological conditions, and because 20% of proteins may be degraded through ubiquitin-independent proteasome pathways under normal or stress conditions, it is likely that these pathways are more important for quotidian function than previously appreciated (Baugh et al., 2009; Ben-Nissan and Sharon, 2014; Pepelnjak et al., 2024). In this review, we focus on ubiquitin-independent proteasomal mechanisms and the emerging role these mechanisms plays in neurodegeneration.

## Search scheme and article selection

PubMed and Google Scholar search engines were first used to identify research articles using search terms including ubiquitin-independent proteasome, neurodegeneration, 20S proteasome, Alzheimer’s Disease, Parkinson’s Disease, Huntington’s Disease, protein aggregation, oxidative stress, PA200, PA28, and aging, among others. However, results from these keyword search terms did not distinguish well between the UPS and ubiquitin-independent mechanisms, so alternative tools employing artificial intelligence (AI) were used. These included Consensus and Semantic Scholar–the primary tools used–as well as Elicit and Research Rabbit. Consensus, Elicit, and Semantic Scholar were leveraged to search databases of >200 million peer-reviewed scientific papers using natural language processing to interpret questions about research topics rather than keywords (e.g., “What roles do ubiquitin-independent proteasome mechanisms play in neurodegenerative disease?” or “Can the 20S proteasome degrade tau without a proteasome activator?”), using machine learning to process the content and context of literature, and using large language models to suggest relevant articles or provide a summary of conflicts and consensus in the literature with references, reducing bias in the search results. After curating a collection of the most relevant articles based on these searches, Research Rabbit was used to visualize connected papers, identifying additional article suggestions. Aside from AI tools, other papers were identified by scanning the references of pertinent articles. After identification, full-text articles published in or before October 2024 were reviewed for ubiquitin-independent proteasome mechanistic relevance. Original articles and reviews were included, and retracted papers were excluded. All articles were peer-reviewed except for one pre-print (indicated in the text). The writing of this review was not AI-generated, and AI tools were used for article identification only.

## Proteasome substrate identification

For decades, evidence has demonstrated that the proteasome has diverse mechanisms of substrate recognition and degradation beyond the canonical UPS pathway (Jariel-Encontre et al., 2008; Baugh et al., 2009; Ben-Nissan and Sharon, 2014; Erales and Coffino, 2014), and a growing number of protein substrates targeted to the proteasome without ubiquitination have been discovered (Rosenberg-Hasson et al., 1989; Bercovich and Kahana, 1993; Coffino, 1998; Sheaff et al., 2000; Bossis et al., 2003; Myers et al., 2018; Makaros et al., 2023). Recent advances include a systematic analysis of human 20S proteasome substrates using a method called proteasomal-induced proteolysis mass spectrometry, developed by Pepelnjak et al. to identify a range of proteins degraded by the ubiquitin-independent 20S (Pepelnjak et al., 2024). Another technique, Global Protein Stability peptidome screening, was developed by Koren et al. (2018) and subsequently applied to identify ubiquitin-independent proteasome substrates (Makaros et al., 2023). These papers and others have demonstrated that proteins central to neurodegeneration, including tau (important in AD and other tauopathies) (David et al., 2002; Grune et al., 2010; Ukmar-Godec et al., 2020), α-synuclein (important in PD and other synucleinopathies) (Tofaris et al., 2001; Nakajima et al., 2005; Alvarez-Castelao et al., 2014; Makaros et al., 2023), huntingtin (important in HD) (Juenemann et al., 2013), as well as many proteins important in stress, transcriptional regulation (like RNA-binding partners and transcription factors), phase granule separation (Myers et al., 2018), and cell cycle regulation (Sheaff et al., 2000; Touitou et al., 2001; Asher et al., 2005; Wiggins et al., 2011), can be degraded through ubiquitin-independent mechanisms (Pepelnjak et al., 2024). These techniques suggest that ubiquitin-independent degradation is far more prevalent than previously believed, although in some cases, more orthogonal approaches or *in vivo* data approximating normal physiology may be needed to definitively support this claim. Although significant advances are rapidly emerging, the exact targeting mechanisms of ubiquitin-independent degradation are still under investigation. However, the 20S proteasome is known to degrade intrinsically disordered proteins (IDPs) like α-synuclein (α-syn) and tau more efficiently than structured proteins (Grune et al., 2010; Alvarez-Castelao et al., 2014; Myers et al., 2018; Ukmar-Godec et al., 2020), and there may be additional specific motifs (Touitou et al., 2001; Bossis et al., 2003; Makaros et al., 2023), including C-terminal degrons (Touitou et al., 2001; Makaros et al., 2023), and structural features including exposed hydrophobic residues (Kisselev et al., 2002), that are recognized for targeted degradation. It has also recently been demonstrated that the 20S can degrade ubiquitinated substrates, degrading the ubiquitin tag along with the protein more quickly than the 26S can deubiquitinate and digest, a mechanism increased during hypoxic stress conditions to clear misfolded/damaged proteins rapidly (Sahu et al., 2021).

To protect the cell from excessive proteolysis, entry into the catalytic chamber of the 20S core is tightly regulated, with its external α-rings partially obstructing the protease active sites (Wenzel and Baumeister, 1995; Groll et al., 2000). To allow substrate entry, 20S proteasomes may interact with pore-opening proteasome activators (PAs) (Knowlton et al., 1997; Hendil et al., 1998; Ortega et al., 2005), or they may allow direct substrate access without a PA (Kisselev et al., 2002). As a standalone molecule, the 20S can recognize and interact with hydrophobic regions of misfolded proteins, which act as degradation signals, as well as IDPs and oxidized proteins (Kisselev et al., 2002; Förster et al., 2003; Raynes et al., 2016; Deshmukh et al., 2023). Because IDPs lack a rigid, well-defined structure, they are more flexible and can more easily enter the narrow entry channel of the 20S proteasome (Suskiewicz et al., 2011), whereas structured proteins require unfolding or linearization by the 19S cap ATPases (Wenzel and Baumeister, 1995; Dong et al., 2019). The independent 20S can undergo conformational changes in its α-rings without ATP hydrolysis that permit self-gated entry of unstructured, oxidized, or misfolded proteins through the narrow entry pore (Kisselev et al., 2002; Förster et al., 2003). This capacity for protein degradation without ubiquitination or energy consumption makes the 20S proteasome uniquely suited to remediate the accumulation of toxic protein aggregates in neurodegenerative diseases, which often cause mitochondrial damage, oxidative stress, and further impairment of the UPS (Ding et al., 2006; Pickering et al., 2010; Li et al., 2011; Huang et al., 2013; Höhn et al., 2020). Importantly, substrate degradation by ubiquitin-independent proteasomal mechanisms, the UPS, or non-proteasomal pathways like autophagy are not necessarily mutually exclusive in the cell, and some substrates may be degraded by one mechanism in some conditions and another mechanism in other conditions, such as oxidative stress (Grune et al., 2010; Suskiewicz et al., 2011; Ben-Nissan and Sharon, 2014; Manfredonia and Kraut, 2022). It is also well-documented that autophagic pathways may be used to clear certain isoforms of these proteins, hypermodified forms, or aggregates, which will not be discussed here (Lee et al., 2013; Watanabe et al., 2020). Increasing research has shed light on how these substrates are targeted and the variety of mechanisms used to facilitate or regulate their degradation through the proteasome.

## Proteasome activators (PAS)

The best-understood mechanisms of substrate targeting are through PAs. Prior research has demonstrated that interaction with 20S molecules require many PAs to use a C-terminal tri-peptide HbYX motif (hydrophobic residue, followed by tyrosine, then any amino acid) that docks into the spaces between ⍺-ring subunits, called 20S ⍺-pockets (Smith et al., 2007; Rabl et al., 2008; Sadre-Bazzaz et al., 2010). In contrast to research on the HbYX motif in archaea models (Smith et al., 2007; Rabl et al., 2008; Yu et al., 2010), recent research shows that human 20S proteasomes, which are hetero-oligomers with seven distinct ⍺-subunits in the outer ⍺-ring rather than the homo-oligomers formed by archaea, may have more heterologous signals, dubbed YΦ motifs by the Gestwicki group in 2022 (Opoku-Nsiah et al., 2022). While HbYX motifs are tripeptides, the YΦ motifs tested were hexapeptide sequences, showing an effect on degradation for each of the last 6 residues of the C-terminus. YΦ refers to Y-F/Y residues at the antepenultimate and penultimate positions at the C-terminus. In addition, as opposed to monovalent PAs like PA200 that require adherence to HbYX/YΦ rules (Sadre-Bazzaz et al., 2010), hetero-oligomeric PAs, which have increased valency due to interactions with multiple ⍺-subunit pockets, allowed some flexibility in C-terminal gating association outside of HbYX/YΦ rules (Opoku-Nsiah et al., 2022). Further research must be performed to catalog the full range of interaction sequences present in human 20S proteasomes, and this may provide insight into regulation of PAs, the importance of any post-translational modifications, and their role in neurodegenerative diseases.

An important implication of these recognition sequences is that they may be useful as drug targets. It has been demonstrated that synthesized HbYX-like peptide mimetics can open the 20S pore and stimulate degradation of unstructured protein substrates, posing a possible therapeutic option for neurodegenerative disease (Chuah et al., 2023). In addition to increasing degradation of tau by the 20S, HbYX mimetics also completely block 20S inhibition by amyloid-β, α-syn, and huntingtin oligomers, further demonstrating the potential for small molecule treatments to restore 20S proteasome activity and increase degradation of disordered substrates prone to aggregation (Chuah et al., 2023).

**FIGURE 1:**
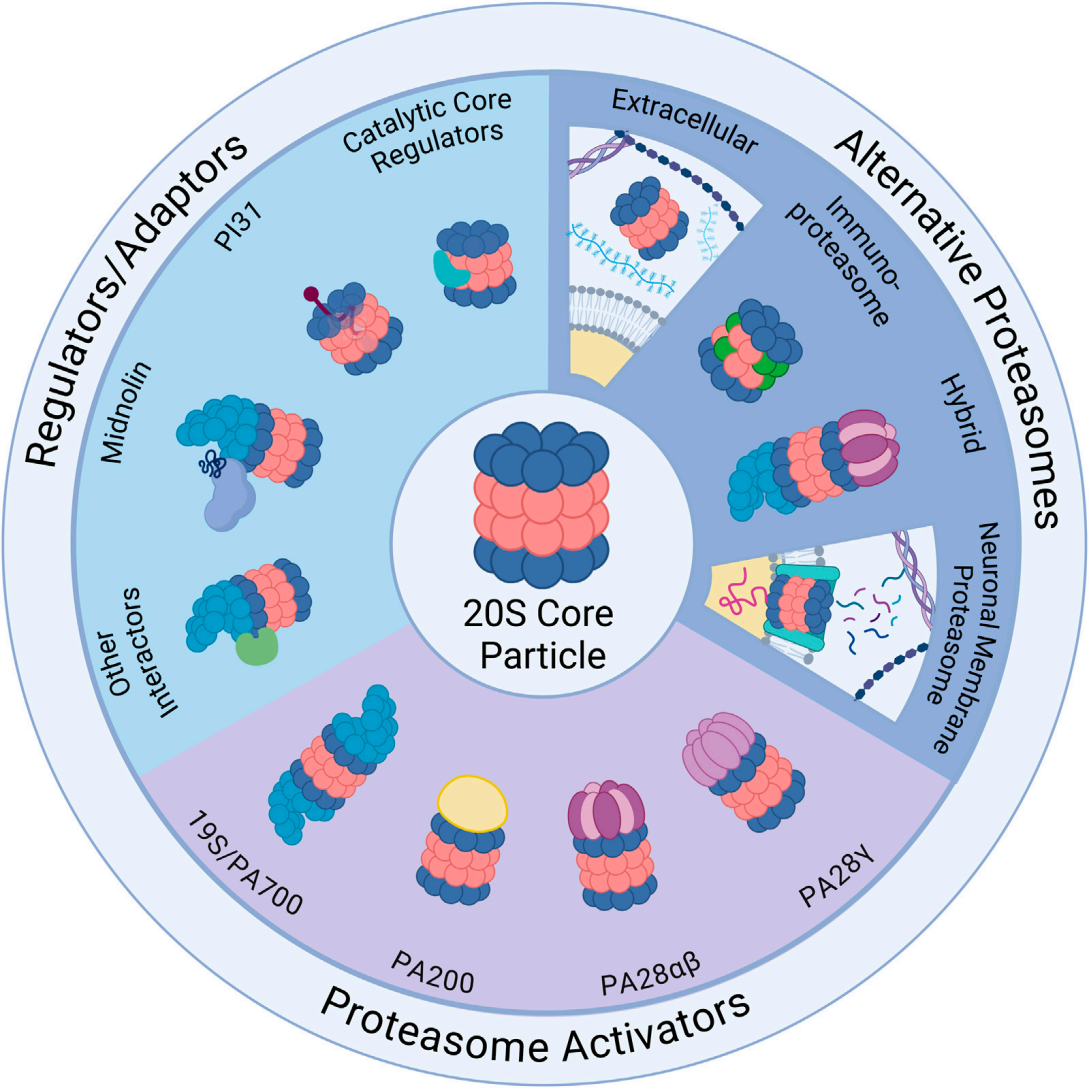
Ubiquitin-Independent Proteasome Complexes and Mechanisms. Illustrated above are the diverse complexes that can form in combination with the 20S core particle (20S) (center illustration). The 20S has 28 subunits that form a barrel structure with 14 α-subunits (blue) and 14 β-subunits (red). *Alternative Proteasomes*: The 20S can be found in various forms including the immunoproteasome, which has alternative β catalytic subunits (green) and can be induced as part of the immune response, and the neuronal membrane proteasome (NMP), which is a neuron-specific proteasome complex localized to the plasma membrane that is used for signaling. The 20S can also be found in the extracellular space and can exist in hybrid forms which have two distinct cap structures on each side of the 20S (shown here with 19S and PA28αβ). *Regulators/Adaptors*: The 20S interacts with several important regulators/adaptors including catalytic core regulators (CCRs), midnolin, PI31, and a broad category encompassing other interactors. Aside from PI31, which interacts with 20S subunits from inside the barrel, most of these regulators associate with the 20S exterior, and in some cases (midnolin and several other interactors) with the 19S-capped 20S. *Proteasome Activators* (PAs): The 20S interacts with a variety of PAs that increase 20S activity by opening the gate formed by α-subunits and permitting substrate entry for degradation. Those pictured include: the 19S cap, which combines with the 20S to form the 26S; PA200, which is monomeric and mainly found in the nucleus; PA28αβ, which is typically cytoplasmic and plays an important role in the immune response; and PA28γ, which has high expression in the brain and serves an important role in cell cycle regulation. Created in BioRender. Church, T. (2025) https://BioRender.com/o73i883.

The most famous PA is the 19S regulatory cap (also called PA700), which in addition to being the canonical activator complex in the UPS has some capacity for facilitating ubiquitin-independent degradation through the 26S proteasome, generating a different set of peptides than 20S alone (Kisselev et al., 1999; Asher et al., 2005; Baugh et al., 2009; Winkler et al., 2013; Ben-Nissan and Sharon, 2014; Tsvetkov et al., 2020). In addition, Tsvetkov et al. showed *in vitro* that when the assembled 26S is stabilized by binding of NADH, an important molecule in aging and metabolism that is sensitive to cellular redox state, it can facilitate degradation of IDPs even in the absence of ATP. However, 20S proteasome catalytic activity was not affected by NADH or NAD+ *in vitro* (Tsvetkov et al., 2020). Other PAs that can bind to the exterior of the 20S core and enhance its activity by inducing central pore opening include PA28 (also called 11S or PSME1) and PA200 (or Blm10 in yeast), both of which use ubiquitin-independent mechanisms to facilitate substrate entry and often target oxidized, unstructured/intrinsically-disordered, or misfolded proteins in specific subcellular locations (Dubiel et al., 1992; Ma et al., 1992; Ustrell et al., 2002; Ortega et al., 2005; Cascio, 2021). In addition to PAs, other major proteasome interactors include PI31 (also called PSMF1), an adaptor protein for proteasome transport in neurons (Liu et al., 2019); midnolin, a regulator of immediate early gene protein and transcription factor degradation (Gu et al., 2023); ECPAS (“Ecm29 proteasome adaptor and scaffold”; also called PSMG1, or Ecm29), a modulator of 26S assembly and disassembly participating in stress responses (Wang et al., 2017; Lee et al., 2020), and catalytic core regulators (CCRs), which allosterically modulate uncapped 20S activity (Deshmukh et al., 2023). These will be explored in greater detail in the following subsections. See Figure 1 and Table 1.

**TABLE 1:** Proteasome nomenclature and subunit-specific links to neurodegenerative disease.

Category	Proteasome complex TYPE	Subclassification	Subunit	Gene	Function and substrates	Neurodegenerative disease associations^a^
20S CP	Constitutive	α type	α1	PSMA6	interaction with proteasome activators (PAs) & regulatory particles (RPs)	PD
			α2	PSMA2	interaction with PAs and RPs	PD
			α3	PSMA4	interaction with PAs and RPs	AD
			α4	PSMA7	interaction with PAs and RPs	PD
			α5	PSMA5	interaction with PAs and RPs	AD, PD
			α6	PSMA1	interaction with PAs and RPs	PD
			α7	PSMA3	interaction with PAs and RPs	PD
			α8	PSMA8	interaction with PAs and RPs	
		β type	β1	PSMB6	Caspase-like degradation; cleaves after acidic/negatively-charged residues	PD
			β2	PSMB7	Trypsin-like degradation; cleaves after basic/positively-charged residues	AD, PD
			β3	PSMB3		
			β4	PSMB2		PD
			β5	PSMB5	Chymotrypsin-like degradation; cleaves after hydrophobic residues, branched amino acids, small neutral amino acids	AD, PD
			β6	PSMB1		
			β7	PSMB4	Interaction site for CCRs	
	Immunoproteasome		β1i	PSMB9, LMP2	Chymotrypsin-like; cleaves after hydrophobic residues, branched amino acids, small neutral amino acids	AD, PD^b^
			β2i	PSMB10, MECL1	Trypsin-like degradation; cleaves after basic/positively-charged residues	AD^b^
			β5i	PSMB8, LMP7	Chymotrypsin-like degradation; cleaves after large hydrophobic residues, branched amino acids, small neutral amino acids	AD^b^
Proteasome activators	PA700 (19S)	AAA+ ATPase	Rpt1	PSMC2	ATPase; base of 19S PA (base)	AD
			Rpt2	PSMC1	ATPase, Gate opening; base	PD
			Rpt3	PSMC4	ATPase, Gate opening; base	PD
			Rpt4	PSMC6	ATPase; base	PD, HD
			Rpt5	PSMC3	ATPase, Gate opening; base	PD
			Rpt6	PSMC5	ATPase; base	AD, PD, HD
Proteasome activators	PA700 (19S)	non-ATPase	Rpn1	PSMD2	PIP scaffold; Ubiquitin receptor (Ub); base	
			Rpn2	PSMD1	PIP scaffold; base	PD
			Rpn3	PSMD3	lid of 19S PA (lid)	
			Rpn5	PSMD12	lid	PD
			Rpn6	PSMD11	lid	AD, PD
			Rpn7	PSMD6	lid	
			Rpn8	PSMD7	lid	PD
			Rpn9	PSMD13	lid	
			Rpn10	PSMD4	Ub receptor; base	
			Rpn11	PSMD14	Deubiquitinase (DUB); lid	
			Rpn12	PSMD8	lid	PD
			Rpn13	ADRM1	Ub receptor, DUB activation; base	
			Rpn15	SHFM1	lid	
	PA28 (11S)		PA28α	PSME1	Gate opening; Chaperone-like function; increased degradation of short peptides	HD^b^
			PA28β	PSME2	Gate opening; Chaperone-like function; increased degradation of short peptides	HD^b^
			PA28γ	PSME3	Gate opening; Allosteric activator; increases trypsin-like activity; targets nuclear proteins	HD^b^
	PA200		PA200	PSME4	Gate opening; increases caspase-like activity	AD, HD^b^
Proteasome regulators/interactors^c^	N/A	Active site regulator	PI31	PSMF1	Proteasome activity regulator; proteasome transport adaptor; interacts with dynein light chain proteins & F-box proteins	AD^b^
		Substrate targeting adaptor	Midnolin	MIDN	Substrate recognition and transport to proteasome; immediate early gene proteins and transcription factors	PD^b^ ^,^ ^e^
		Catalytic core regulators^d^	DJ-1	PARK7	Allosteric Inhibitor; Nrf2 pathway activator	PD^b^
			NQO1	NQO1	Allosteric inhibitor; oxidoreductase; quinone detoxification	AD^b^
		Other adaptors^c^	ECM29/ ECPAS	ECPAS	20S-19S uncoupling; adaptor and scaffold	

^a^ Modified from a 2021 paper by Fernández-Cruz and Reynaud (2021).

^b^ Additional sources: β1i β2i β5i (Mishto et al., 2006; Aso et al., 2012; Orre et al., 2013; Orre et al., 2014; Yeo et al., 2019; Park et al., 2024); PA28γ (Seo et al., 2007; Jeon et al., 2016; Cascio, 2021); PA28αβ (Geijtenbeek et al., 2022; Kriachkov et al., 2023); PA200 (Dange et al., 2011; Aladdin et al., 2020); PI31 (Sherva et al., 2011); midnolin (Obara et al., 2017; Obara and Ishii, 2018); DJ-1 (Moscovitz et al., 2015); NQO1 (Bian et al., 2008; Tsvetkov et al., 2011).

^c^ This table includes examples of proteasome interactors and regulators but is not an exhaustive list. Many other interactors exist in cells.

^d^ Catalytic Core Regulators (CCRs) represent a family of proteasome interactors. Listed here are two CCRs, shown to have relevance in neurodegenerative disease.

^e^ Controversial result; evidence against (Billingsley et al., 2020).

The table outlines the nomenclature for mammalian proteasome complexes, grouping subtypes by color. Note the specific gene names for several of the β-subunits do not align intuitively with the protein name. Also included are several subunit-specific roles within the proteasome complex. The final column is modified from a 2021 paper by Fernández-Cruz and Reynaud (2021), referencing individual subunits linked specifically to the neurodegenerative diseases discussed in this review. PA200, PA28α, PA28β, PA28γ, midnolin, PI31, DJ-1, and NQO1 are referenced from different sources, and additional sources are added for immunoproteasome subunits β1i, β2i, and β5i. PIP, proteasome interacting protein; CCR, catalytic core regulator. DUB, deubiquitinating enzyme.

### PA28

PA28 is a heptameric, ring-shaped, and ATP- and ubiquitin-independent 20S PA that promotes rapid degradation of small, unstructured protein fragments, short peptides, and oxidized or misfolded proteins in the nucleus and cytoplasm by binding to the ends of the 20S core and inducing conformational changes that widen the 20S pore (Ma et al., 1992; Knowlton et al., 1997; Zhang et al., 1999; Thomas and Smith, 2022). Importantly, there are multiple isoforms of PA28, including PA28α (also called REGα and PSME1), PA28β (also called REGβ and PSME2), and PA28γ (also called REGγ and PSME3), which have distinct sets of functions and substrates (Knowlton et al., 1997; Thomas and Smith, 2022). PA28α and PA28β are mainly cytoplasmic and typically combine to form heteroheptamers, but PA28α is expressed at higher levels in the brain than PA28β and can form a homoheptamer (Knowlton et al., 1997; Zhang et al., 1999; Noda et al., 2000). PA28γ also forms homoheptamers and is primarily nuclear, ubiquitously expressed in all organ systems, with particularly high expression in the brain (Noda et al., 2000; Cascio, 2021; Frayssinhes et al., 2021). PA28γ is an interferon-γ- (IFNγ) and ubiquitin-independent PA which serves as a regulator of DNA replication, DNA repair, transcription, cell cycle control, and p53 tumor suppressor stability (Zhang and Zhang, 2008).

Because it is not an ATPase, PA28 cannot unfold proteins and has a preference for disordered or partially unfolded proteins, which can enter the 20S catalytic chamber without additional unfolding (Frayssinhes et al., 2021). It can interact with both the typical 20S core (constitutive 20S) and the immunoproteasome (a modified, inducible complex described in the “*Immunoproteasome*” section of this review), inducing different allosteric effects on each (Lesne et al., 2020). Indeed, PA28 becomes increasingly important in aging and neurodegenerative disease, when the UPS is compromised and damaged proteins accumulate (Seo et al., 2007), through its regulation of 20S activity, its ability to activate 26S as a hybrid proteasome (19S-20S-11S/PA28) (Tanahashi et al., 2000), and as a standalone chaperone-like molecule and chaperone regulator (Minami et al., 2000; Adelöf et al., 2018; Adelöf et al., 2021). PA28 expression is also upregulated under conditions of high protein damage including oxidative stress, indicating an important role in maintaining proteostasis by mitigating oxidative damage, and it often accompanies upregulation of the immunoproteasome (Pickering et al., 2010; Pickering et al., 2012).

While best characterized in other cell types, studies in neurons have shown that PA28 promotes ubiquitin-independent proteasomal degradation of oxidized and misfolded proteins and protects against oxidative stress (Li et al., 2011; Pickering and Davies, 2012), increasing evidence for its role as a 20S regulator in oxidatively-burdened neurodegenerative disease states. In addition, PA28⍺*β* overexpression showed sex-specific benefits for female mice in preventing age-related protein aggregation, hypothesized by the authors to be a novel, proteasome-independent, chaperone-like function (Adelöf et al., 2018). Moreover, PA28⍺*β*, plays major roles in the immune system through regulation of the immunoproteasome, described in the “*Immunoproteasome*” section below. In neurons and microglia, exposure to cytokine IFNγ or other pro-inflammatory factors during an immune or inflammatory response increases PA28αβ expression and its association with the immunoproteasome (Rivett et al., 2001; Pickering et al., 2010; Pintado et al., 2012). Because neuroinflammation is increasingly recognized as a contributor to the development of neurodegenerative diseases, dysregulation of PA28αβ - and therefore the immunoproteasome - can contribute to neuroinflammatory processes through neurons and glia (Leng and Edison, 2021; Malek et al., 2024). Notably, studies have also reported altered PA28 expression in Alzheimer’s disease brains (Krzyzanowska et al., 2015) and PA28γ may play a complex role in the etiology of HD, which will be described in the “*Huntington’s Disease*” section below (Cascio, 2021). Expansion on the significance of PA28 in specific neurodegenerative disease will be included in sections below.

### PA200

PA200 is a large, monomeric, and ATP- and ubiquitin-independent 20S proteasome activator found predominantly in the nucleus which associates with the 20S core and regulates DNA repair mechanisms, transcription, and the cell cycle through targeted, acetylation-dependent degradation of histones and other protein targets (Ustrell et al., 2002). Its structure has two apertures for substrate entry and forms a dome-like cap on the 20S to open it (Guan et al., 2020). Some evidence suggests PA200 alters the relative activity of the 20S β catalytic subunits, increasing β1 (Ustrell et al., 2002) or β2 (Toste Rêgo and Da Fonseca, 2019) activity compared to the uncapped 20S.

Because PA200 does not have ATPase activity, it primarily acts on peptides and disordered and partially unfolded proteins, although there is a possibility it has some intrinsic unfolding ability through recruitment of other factors or conformational changes of substrates. In addition to possible regulatory roles in proteasome stability or maturation (VerPlank et al., 2024), PA200 is upregulated in response to DNA damage and induces opening of the α-ring substrate entry channel of the 20S, allowing for rapid clearance of oxidized, aggregated, and misfolded substrates (Ortega et al., 2005). These substrates include tau (Dange et al., 2011) and N-terminal huntingtin protein fragments (Aladdin et al., 2020), the proteins responsible for the pathogenic aggregates in AD and HD, respectively. Because research on PA200 function in the nervous system is still limited, its role in neurodegenerative diseases, its regulation and interactions with other PAs, and its cell type-specific characteristics in neurons remain mostly unknown. In fact, depending on the disease state, PA200 may ameliorate or worsen neurodegeneration in *in vivo* disease models (Aladdin et al., 2020; VerPlank et al., 2024).

### Additional cap conformations and hybrid proteasomes

Further research is being performed to investigate the existence and roles of additional alternative caps, including hybrid proteasomes with different caps (e.g., one 19S and one alternative cap associated with a 20S core) (Hendil et al., 1998; Tanahashi et al., 2000; Cascio et al., 2002). While initially thought to be absent from the brain (Noda et al., 2000), along with PA28*β*, more recent data have demonstrated the presence of hybrid proteasomes and PA28*β* (Adelöf et al., 2018; Kriachkov et al., 2023). The significance of hybrid proteasomes in neurons is not yet well understood, but they may provide finely calibrated regulation of substrates or alter proteasome catalytic activity to generate a different set of peptides, as has been seen in the immune system to modify peptide products for antigen presentation (Hendil et al., 1998; Cascio et al., 2002). Hybrid proteasomes have also been proposed to use the 19S for protein unfolding and entry into the 20S chamber and PA28 for the rapid release of digestion products (Pratt and Rechsteiner, 2008; Kriachkov et al., 2023). It is unknown if PI31 or other adaptors are involved in hybrid proteasomes.

In addition, there may be tissue-specific, ubiquitin-independent alternative caps, as has been noted for ubiquitin-dependent PAs (Goldberg et al., 2021), or transient caps that do not assemble into stable structures like PA28 or PA200 but which interact briefly with the 20S core to modulate its activity or substrate channel access (Clemen et al., 2015; Esaki et al., 2018; Goldberg et al., 2021). These phenomena may be especially likely in neurons, which have specialized proteasomes, unique activators, and additional proteasomal physiological functions (Bingol and Schuman, 2005; Ramachandran and Margolis, 2017; Goldberg et al., 2021). An example of this is the characterization of neurodegenerative disease associated protein valosin-containing protein (also called Cdc48, TER94, and p97), a HbYX motif-containing PA which plays a role in ubiquitin-dependent degradation (Johnson et al., 2010; Esaki et al., 2018). In addition, some evidence suggests that 19S caps may not require ubiquitin for degradation of some substrates (Kisselev et al., 1999; Winkler et al., 2013; Tsvetkov et al., 2020). The full range of endogenous proteasome interactors and alternative caps are still being explored, and more molecules are likely to emerge.

## Proteasome regulators

In addition to PAs, other proteasome adaptor and interactor proteins can regulate proteasome assembly and disassembly, link the proteasome to signaling pathways, regulate substrate specificity, and direct intracellular trafficking of proteasomes. The functions of these adaptors vary by cellular conditions, cell type, and activation of intersecting regulatory pathways (Arkinson et al., 2024). See Figure 1 and Table 1.

### PI31

While not a PA, PI31 has been proposed as an endogenous 20S proteasome regulator and is targeted to the 20S via a HbYX motif. *In vitro*, it interacts with both 20S and 26S constitutive proteasomes and has been found to inhibit the 20S and to prevent binding of the 19S cap and PA28 (McCutchen-Maloney et al., 2000; Li et al., 2014; Liu et al., 2019; Wang et al., 2024). In contrast to the constitutive 20S and 26S, immunoproteasomes are capable of cleaving the PI31 C-terminus, preventing its binding and its inhibition of the core catalytic subunits (Wang et al., 2024). Separately, another study suggests PI31 affects immunoproteasome assembly (Zaiss et al., 2002). Although much of the research regarding PI31 has been performed *in vitro* (Li et al., 2014; Wang et al., 2024), *in vivo* experiments have contributed to a complex picture of PI31-mediated proteasome regulation. In a more physiological context, PI31 may in fact activate proteasome degradation through the 26S, and in addition, both knockout and overexpression of PI31 are lethal, indicating that cells are sensitive to PI31 amount (Bader et al., 2011). *In vivo* experiments in mouse motor neurons have demonstrated that PI31 acts as a proteasome regulator and adaptor protein that connects the proteasome to transport machinery for translocation down neuronal projections including axons, an essential function for maintaining a healthy proteasome supply to diverse cellular locations for their various functions (Bader et al., 2011; Liu et al., 2019). Loss of PI31 contributes to neurodegeneration, as its regulatory activity is required for normal proteasome function, maintenance of synapses, and neuronal survival (Bader et al., 2011; Liu et al., 2019; Minis et al., 2019). Genome-wide association studies have linked PI31 to AD risk (Sherva et al., 2011), and a direct antagonist of PI31, called valosin-containing protein (VCP), causes a familial type of the neurodegenerative disorder amyotrophic lateral sclerosis (Johnson et al., 2010; Clemen et al., 2015).

### Catalytic core regulators (CCRs)

As the importance of non-UPS proteasome activity is becoming more apparent, it is increasingly critical to study regulators of these mechanisms. A newly discovered family of multi-functional regulatory proteins that directly interact with the 20S core to closely modulate its cap-independent degradation of IDPs and damaged, partially-unfolded proteins are the Catalytic Core Regulators (CCRs) (Olshina et al., 2020; Deshmukh et al., 2023). These CCRs are allosteric regulators with shared structural features including a common N-terminal sequence motif and a Rossman fold, providing further evidence that HbYX motifs represent only a portion of the structural features characterizing proteasome regulators. CCRs bind to the external surface of the 20S *β*7 (PSMB4) subunit and induce a conformational change that inhibits all three catalytic mechanisms of degradation without plugging the substrate entry gate and can protect substrates from degradation, including ⍺-syn, which forms toxic oligomers in PD and other synucleinopathies (Deshmukh et al., 2023). CCRs are critical for coordinating the oxidative stress response through interaction with transcription factor Nrf2 and activation of a range of response factors including upregulation of 20S subunits (Olshina et al., 2020; Deshmukh et al., 2023). The identification of the structural features underlying allosteric regulation of degradation by the 20S also provides insight relevant to the development of selective, synthesized inhibitors of 20S proteasomes and possible therapeutic options for neurodegenerative diseases like PD, which is directly affected by a CCR called DJ-1 and is discussed later in this review (Moscovitz et al., 2015).

### Midnolin

Midnolin is an inducible, chaperone-like protein that associates with the 26S proteasome to promote selective, ubiquitin-independent degradation of transcription factors and other nuclear proteins (Gu et al., 2023). Recent studies indicate its targeting mechanism uses an internally symmetrical “catch” domain that induces a conformational change in unstructured regions of protein substrates to capture them for destruction by the proteasome. Midnolin associates with the proteasome using a C-terminal ⍺ helix, which does not contain a HbYX motif or YΦ motif, through an unknown mechanism, and it facilitates degradation of targets bound to the catch domain using an N-terminal ubiquitin-like domain. While the reasons for its preference for the 26S over the 20S are not well understood, midnolin co-immunoprecipitates with both 19S and 20S subunits (Gu et al., 2023).

Substrates of midnolin include transcriptional regulators and immediate early gene products, which are rapidly induced upon stimulation by a variety of stimuli and regulate transcription of longer-term sets of proteins in response to a particular stimulus (Chiba et al., 2024; Gu et al., 2023). Notably, midnolin and some of its substrates have been identified as PD risk genes, with deletion of midnolin resulting in loss of parkin expression, increased expression of ⍺-syn, and induction of PD phenotypes including loss of neurite outgrowth (Obara et al., 2017; Obara and Ishii, 2018). Obara et al. used microarray analysis to show that 10.5% of sporadic PD patients and 0% of healthy controls lack one copy of midnolin, positing a role for midnolin loss in development of PD (Obara et al., 2017). These data were supported by another large cohort study by the same research team in 2019, which showed a significant odds ratio of 4.35 with midnolin copy number loss for development of PD, with the odds ratio increasing to 22.3 when copy number loss is defined by large deletions (Obara et al., 2019). However, using whole genome sequencing and analysis of a public database of structural variants, Billingsley et al. and the International Parkinson’s Genomics Consortium did not identify PD-associated midnolin deletions and disputed the determination of midnolin as a PD risk gene, indicating that further study with orthogonal methods is required to investigate midnolin association with PD and resolve controversy (Billingsley et al., 2020).

### Additional regulators/adaptors

Beyond the above regulators, there are other critical 20S interactors and adaptors that affect 20S activity and assembly briefly described here. Chaperones PAC1-PAC4 and POMP are crucial for the proper formation and maturation of the proteasome from its constituent monomeric subunits (Hirano et al., 2005; Hirano et al., 2006; Fricke et al., 2007; Le Tallec et al., 2007), and adaptors including ECPAS contribute to 26S assembly and disassembly *in vivo* (Wang et al., 2017; Choi et al., 2023). The ECPAS-20S interaction regulates the 20S:26S ratio and modulates the balance between ubiquitin-dependent and -independent mechanisms for adaptation to conditions like glucose deprivation or oxidative stress, in which it facilitates disassembly of 26S to 20S to support degradation of oxidized and misfolded substrates (Leggett et al., 2002; Wang et al., 2017; Choi et al., 2023). Interactions among the proteasome, ECPAS, and ankyrin G also regulate critical remodeling of the axon initial segment of neurons, found to have significant structural abnormalities in AD-affected neurons (Lee et al., 2020).

In addition to proteins directly bound to the 20S, there are also substrate-bound proteins critical to its regulation, deemed “nanny” proteins, that protect newly synthesized intrinsically disordered 20S substrates from degradation and allow new intrinsically disordered proteins (IDPs) to mature (Tsvetkov et al., 2009). Potential nanny proteins suggested by Tsvetkov et al., have been linked to a variety of nervous system disorders (Enokido et al., 2010; Kamińska et al., 2024; Yuhan et al., 2024). Conversely, chaperones like Hsp70 and Hsp110, dysfunction of which has been linked to neurodegenerative diseases, facilitate the targeting of substrates to the proteasome for ubiquitin-dependent and ubiquitin-independent degradation (Eroglu et al., 2010; Turturici et al., 2011; Hjerpe et al., 2016; Kandasamy and Andréasson, 2018; Taguchi et al., 2019; Vinokurov et al., 2024). Hsp70 has further been demonstrated to interact with ubiquilin2, a shuttling factor that brings substrates to the proteasome and plays a role in neurodegeneration (Wang et al., 2006; Zhang et al., 2014; Hjerpe et al., 2016; Ma et al., 2023). While members of the ubiquilin family typically require ubiquitin for trafficking proteasome degradative targets (Zhang et al., 2014; Itakura et al., 2016), Makaros et al. demonstrated that ubiquilins may also mediate ubiquitin-independent proteasome substrate identification (Makaros et al., 2023).

There are likely many additional yet-uncharacterized proteins that regulate 20S proteasome activity in neurons. In addition, post-translational modifications like phosphorylation, oxidation, or acetylation can also alter proteasome activity and 20S interaction with regulators in various cell types, especially as cells age (Bulteau et al., 2000; Bulteau et al., 2001; Ishii et al., 2005; Kors et al., 2019).

## Specialized proteasomes

See Figure 1 and Table 1.

### Immunoproteasome

When stimulated by interferon-γ (IFNγ) or oxidative stress, immune cells and some other cell types (e.g., microglia in the nervous system (Orre et al., 2013; Malek et al., 2024)) can produce a modified proteasome, the immunoproteasome, which can act through ubiquitin independent or ubiquitin dependent mechanisms and replaces the three catalytic β-subunits (β1, β2, β5) in the constitutive proteasome core with three unique catalytic subunits (β1i, β2i, β5i, also called PSMB9/LMP2, PSMB10/MECL-1, PSMB8/LMP7) (Noda et al., 2000; Basler et al., 2013; Freudenburg et al., 2013; Rock et al., 2014; Johnston-Carey et al., 2015; Ettari et al., 2017; Winter et al., 2017; Abi Habib et al., 2022). This subunit replacement allows for the generation of longer peptides which can be further processed and presented as antigens that allow cells to determine self vs. non-self, an important component of immune responses, although an increasing number of roles for the immunoproteasome are being recognized (Pickering et al., 2010; Abi Habib et al., 2020; Tundo et al., 2023). It appears to be particularly important for the clearance of oxidized and misfolded proteins in response to oxidative stress (Pickering et al., 2010; Pickering and Davies, 2012). Immunoproteasomes are upregulated in reactive glia in AD mouse models (Orre et al., 2013; Orre et al., 2014), which may be critical for elimination of misfolded or damaged proteins that could spread between cells and cause disease progression. Studies using immunoproteasome-specific inhibitors demonstrate improvements in cognitive decline in AD mice, showing that the immunoproteasome may contribute to pathology in association with neurodegeneration (Yeo et al., 2019; Park et al., 2024). The ability to generate highly selective inhibitors for modified immunoproteasome subunits provides a pathway to evaluate the effects of these complexes without affecting constitutive proteasome activity, a therapy that could have benefits in neurodegenerative diseases impacted by neuroinflammation (Johnston-Carey et al., 2015; Malek et al., 2024).

While the expression of immunoproteasome in the young and healthy brain is very low or negligible, and for a long time it was believed that the immunoproteasome was not expressed in the brain at all (Noda et al., 2000), immunoproteasome has been detected in both neurons and glia in aged healthy brains and brains with neurodegeneration (Diaz-Hernandez et al., 2003; Mishto et al., 2006; Aso et al., 2012; Ugras et al., 2018), suggesting that the induction of immunoproteasome in the brain may be a result of aging, neurodegeneration, or neuroinflammation. Neuroinflammation can exacerbate neurodegeneration but requires additional impaired proteasomal degradation to induce disease phenotypes (Pintado et al., 2012; Malek et al., 2024). Indeed, proteasome research in AD brains has demonstrated a notable induction of immunoproteasome subunits and changes in proteasome activity and composition, although the specific changes observed have varied substantially across studies (e.g., a decrease of only trypsin-like activity vs decreases in both chymotrypsin-like and caspase-like activity; a decrease in β1 expression and a proportional increase in β1i/LMP2 expression vs little change in expression levels) (Mishto et al., 2006; Aso et al., 2012; Keller et al., 2000a; Davidson and Pickering, 2023). In contrast to findings showing decreased activity, a recent study using advanced activity-based probes to detect global proteasome activity in human AD brain tissue detected elevated activity (Türker et al., 2023).

An induction of immunoproteasome has also been detected in HD brains and PD brains (Diaz-Hernandez et al., 2003; Ugras et al., 2018). Concurrent with an increase in LMP2 and LMP7 expression, an increase in trypsin- and chymotrypsin-like activity was observed in HD brains in the areas most affected (Diaz-Hernandez et al., 2003), and in PD brains, an increase in expression of immunoproteasome subunit LMP7 (β5i) was observed (Ugras et al., 2018). A recent study in mice found that knocking out immunoproteasome in brain can also cause seizures, tau hyperphosphorylation, increased polyubiquitination, and neurodegeneration, and the authors suggest that immunoproteasome has a role in healthy brain aging (Leister et al., 2024). In-depth descriptions of the immunoproteasome in neurodegeneration can be found in other recent reviews (Zerfas et al., 2020; Tundo et al., 2023).

### Neuronal membrane proteasome

A specialized proteasome found in the plasma membrane of neurons, called the neuronal membrane proteasome (NMP), is another form of 20S proteasome that functions through ubiquitin-independent mechanisms (Ramachandran and Margolis, 2017). The NMP degrades nascent polypeptide chains from ribosomes closely associated with the membrane to form its signaling molecules (Ramachandran et al., 2018), but it is not yet known how substrate selection or recognition motifs to the NMP may differ from other 20S proteasomes in neurons. Unlike proteasomes whose primary role is in protein turnover, the NMP degrades intracellular proteins into peptides expelled into the extracellular space, creating small, specific peptide signaling molecules that serve additional functions in neurons that are important in synaptic regulation, including NMDA receptor activity modulation, and in pain sensation modulation (Ramachandran and Margolis, 2017; Ramachandran et al., 2018; Türker et al., 2024; Villalón Landeros et al., 2024). Because the NMP regulates neuronal circuits and is essential in learning-induced behavioral plasticity (He et al., 2023), it is possible that dysfunction of the 20S proteasome induced by aging and proteotoxic aggregates in neurodegeneration may also disrupt NMP function, further contributing to declining cognition in neurodegenerative diseases. Supporting this hypothesis, a preprint in bioRxiv by Paradise et al. showed an association between NMP and ApoE, a critical AD risk gene (Paradise et al., 2023). In their experiments, NMP co-purified with ApoE, suggesting a physical interaction, and inhibition of the NMP was sufficient to cause aggregation of newly-synthesized tau. Future studies will reveal the full details of this novel proteasome complex and its function in brain health and disease.

### Extracellular proteasome

While proteasomes are typically thought of as intracellular, increasing evidence has shown 20S proteasomes in extracellular vesicles (EVs) and free-floating in a variety of body fluids, including the interstitial fluid and the cerebrospinal fluid of the brain (Mueller et al., 2012; Ben-Nissan et al., 2022). It has been reported that extracellular proteasomes rarely contain 19S or PA200 PAs (Kulichkova et al., 2017; Tsimokha et al., 2020), although this is debated and may be body fluid/tissue specific (Ben-Nissan et al., 2022). However, they are often free floating as 20S or accompanied by PA28αβ or PI31, and they have significant levels of post-translational modifications, including several unique from other proteasome complexes (Tsimokha et al., 2020; Ben-Nissan et al., 2022), which may affect their function or localization to the extracellular space. The mechanisms of extracellular release and the source of these proteasomes are not yet fully understood, but data have shown release of 20S core particles and PA28 molecules by immune cells in microparticles subject to later dissolution (Bochmann et al., 2014; Bonhoure et al., 2022). It is possible that neurons or glia could release proteasomes as part of normal physiology or as part of a stress response (Ben-Nissan et al., 2022), or that cell damage or cell death causes intracellular proteasomes to leak into the extracellular space. As extracellular proteasomes have been shown to participate in a variety of cellular functions across cell types and disease states (Ben-Nissan et al., 2022), they could serve important roles in regulating protein clearance, especially of IDPs and damaged or oxidized proteins (Bonhoure et al., 2022), or in reducing neuroinflammation (Dianzani et al., 2019) by degrading pro-inflammatory cytokines in the extracellular milieu in the nervous system. Indeed, Dianzani et al. demonstrated that extracellular proteasomes can generate functional peptides and play a role in regulating cell migration and inflammation through cleavage of extracellular osteopontin, a cytokine implicated in diseases like multiple sclerosis (Dianzani et al., 2017; Dianzani et al., 2019).

Notably, early data has suggested differential regulation and expression of extracellular proteasomes and proteasome regulators found in EVs in neurodegenerative diseases including PD, raising the possibility of using proteasomal changes in EVs as a biomarker (Wang et al., 2019; Thompson et al., 2020). These results are encouraging for development of screening tools for neurogenerative disease and require more follow-up study. Critical questions about their functions remain, from regulation of their release to their role in healthy *versus* diseased brains (Dwivedi et al., 2021). While still in early stages, study of extracellular proteasomes in neurodegeneration has significant promise for understanding disease etiology and identifying novel therapies, including possibly for degradation of extracellular aggregates like amyloid-β plaques. The development of standardized, reliable tools for measuring their activity in the brain or spinal cord are needed. This recent review by Ben-Nissan et al. (2022) discusses the current understanding of the extracellular proteasome in depth, as well as experimental strategies to study this increasingly-recognized molecule.

## Aging, oxidative stress, and protein aggregation

**FIGURE 2:**
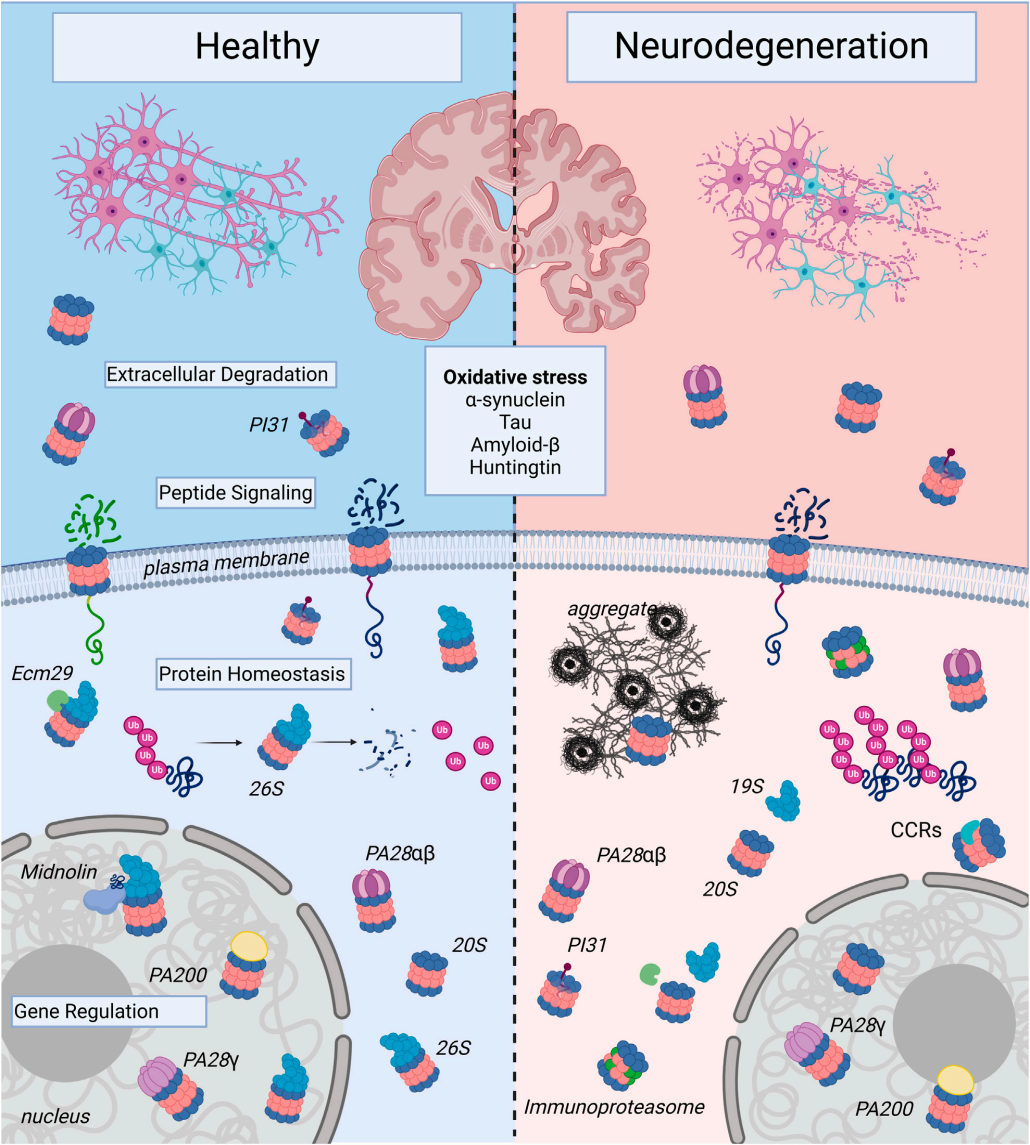
Proteasome Complexes in Health and Neurodegenerative Disease: The illustration shows healthy neurons and neurons affected by neurodegenerative disease. Note, the neurons undergoing neurodegeneration show fragmentation to illustrate decreased survival, which contributes to the decline in memory, cognition, and motor control seen in age-associated neurodegenerative illnesses such as Alzheimer’s Disease, Huntington’s Disease, and Parkinson’s Disease. At the molecular level, this inevitable cell death occurs in part due to proteasome dysregulation and is triggered by oxidative stress and toxic forms of aggregate-forming proteins such as α-synuclein, tau, amyloid-β, and huntingtin. In healthy neurons, there are a variety of proteasome complexes that exist under physiological conditions. In the nucleus, proteasomes with different proteasome activators (PAs; PA200, PA28γ, etc.) or uncapped 20S core particles (20S) have been demonstrated to regulate gene transcription. Cytosolic 20S proteasomes can also be activator-associated (e.g., 26S, PA28αβ) or uncapped. 26S proteasomes can mediate degradation of ubiquitinated proteins and produce small peptide fragments and free ubiquitin (Ub). In the extracellular space, there can be free as well as PA-capped 20S proteasomes, and in neurons, the 20S can be localized to the plasma membrane, where it serves a signaling function. In neurodegenerative diseases, proteasomes shift from predominantly ubiquitin-dependent degradation through the 26S to ubiquitin-independent degradation through alternatively-capped or uncapped 20S complexes. In addition, expression of immunoproteasome subunits is induced, particularly in the setting of chronic neuroinflammation. Proteasome complexes are regulated by adaptors like Ecm29, which mediates assembly and disassembly of the 26S proteasome from the 19S and 20S); PI31, a regulatory molecule that modulates proteasome activity from inside the 20S core; and catalytic core regulators (CCRs), which allosterically regulate the 20S to protect vital intrinsically-disordered proteins from degradation and serve critical roles in the oxidative stress response. Proteasomes are complex and heterogeneous molecules, and targeting different forms of the proteasome may prove useful for the development of preventative and disease-modifying therapies in neurodegeneration, an active area of research. Created in BioRender. Church, T. (2025) https://BioRender.com/k79b247.

See Figure 2 and Table 1.

### Aging

As neurons age, damage to the proteome increases and overall neuronal proteasome activity decreases (Davidson and Pickering, 2023). Both the UPS and ubiquitin-independent proteasome function decline, further contributing to the aggregation of neurodegeneration-associated proteins such as tau, amyloid-β (Aβ), and α-syn (Bulteau et al., 2000; Keller et al., 2000b; Ding et al., 2006; Kelmer Sacramento et al., 2020; Cuanalo-Contreras et al., 2023; Davidson and Pickering, 2023). In addition, while glia normally secrete chaperones that assist neurons in maintaining proper protein folding, age-related 20S dysfunction could disrupt this process and further contribute to a decline in neuronal proteostasis (Chaplot et al., 2020; Leng and Edison, 2021).

With age, there is a shift from 26S to 20S proteasome activity concurrent with the decline in proteasome function, causing a relative increase in 20S activity (Keller et al., 2000b; Ding et al., 2006; Tonoki et al., 2009; Choi et al., 2023; Davidson and Pickering, 2023; Türker et al., 2023). Factors that contribute to this change include: oxidative stress, calpain activation, impaired assembly and recycling, and increased demand for IDP degradation (Huang et al., 2013; Coskuner-Weber et al., 2022; Davidson and Pickering, 2023). As aging is associated with increased oxidative stress and a decline in mitochondrial function, oxidation and damage to proteasome subunits can occur, especially subunits of the 19S cap (Reinheckel et al., 1998; Huang et al., 2013). Because 26S activity, assembly, and recycling are ATP-dependent, dysfunction of mitochondria also limits energy availability, further impairing 26S function and turnover. Furthermore, as oxidative damage accumulates, NADH, which stabilizes the 26S, oxidizes to its NAD+ form, compounding 19S dissociation from the 20S (Tsvetkov et al., 2014). As 26S activity declines, the 20S, which is more resilient to oxidative damage (Reinheckel et al., 1998), becomes more active in neurons by comparison, and its relative levels increase as association with the 19S cap decreases (Huang et al., 2013). Calpains, which are calcium-dependent proteases activated in aging and neurodegenerative diseases, cleave and inactive Rpn10, a 19S subunit, further decreasing 26S activity (Huang et al., 2013). Because aggregation-prone IDPs and damaged proteins with intrinsically disordered regions (IDRs) are preferentially degraded by the 20S rather than 26S, this shift to 20S activity may help to counteract the buildup of toxic protein aggregates over time in aging neurons (Opoku-Nsiah and Gestwicki, 2018; Davidson and Pickering, 2023).

### Oxidative stress

In aging and neurodegenerative disease, a snowball effect of increasing oxidative stress, production of damaged and oxidized proteins, and inhibited proteasome activity can contribute to a progressively worsening cycle leading to neuronal dysfunction and cytotoxicity (Davies, 2001; Raynes et al., 2016). Sulfhydryl groups of the 19S PA are especially vulnerable to oxidation, causing 19S caps to lose their capacity to facilitate proteolysis and resulting in 26S disassembly by Ecm29/ECPAS as an adaptive response to oxidative stress (Shringarpure et al., 2001; Choi et al., 2023; Arkinson et al., 2024). 19S caps are sequestered by Hsp70 during the oxidative insult (Grune et al., 2011), and 20S subunits, immunoproteasome, and PA28αβ are upregulated (Pickering et al., 2010; Moscovitz et al., 2015; Davidson and Pickering, 2023). Because of the shift to favor 20S-mediated protein degradation during oxidative stress, 20S proteasomes are responsible for the majority of proteasome-mediated degradation in these conditions and do not require ubiquitin (Shringarpure et al., 2001; Ding et al., 2006; Raynes et al., 2016). Additionally, while transient mild oxidative stress increases ubiquitin activating/conjugating activity, this activity decreases during sustained oxidative stress (Shang and Taylor, 2011). Because the 20S proteasome does not require a ubiquitin tag to identify substrates, its degradation of substrates must be regulated to destroy harmful proteins while protecting IDPs and IDRs important for the oxidative stress response and normal cellular functions like cell cycle regulators, tumor suppressors, and signaling proteins. There are diverse posttranslational modifications that can regulate the relative activities of different proteasome species, including under oxidative stress conditions, but additional robust protective pathways are required. Several of these regulatory mechanisms (Bi et al., 2021), including catalytic core regulators (CCRs; see *“Catalytic Core Regulators*” section above) and PI31-mediated activity modulation, have only recently been uncovered, and investigation continues (Liu et al., 2019; Minis et al., 2019; Olshina et al., 2020; Deshmukh et al., 2023).

A necessary antioxidant pathway that emphasizes the role of ubiquitin-independent proteasome degradation involves the 20S proteasome, CCRs DJ-1 and NQO1, and transcription factor Nrf2 (nuclear factor E2-related factor 2) (Pickering and Davies, 2012). DJ-1 (also called PARK7) is a critical regulatory protein that stabilizes Nrf2 under oxidative stress conditions. Nrf2 translocates to the nucleus, where it upregulates a variety of proteins important in antioxidant defense, including subunits of the 20S proteasome and PA28⍺*β*, but not the immunoproteasome (which is induced by a different mechanism) or 19S subunit (Clements et al., 2006; Pickering and Davies, 2012). Nrf2 also induces the expression of NQO1 (NAD(P)H quinone oxidoreductase 1), an enzyme which prevents reactive oxygen species formation and acts as a sensor for cellular redox state, avoiding degradation during oxidative stress but being rapidly degraded as cellular conditions normalize (Moscovitz et al., 2012; Yuhan et al., 2024). In addition, both DJ-1 and NQO1 are CCRs and act allosterically to inhibit the 20S, thus rescuing partially unfolded proteins from degradation, an important protective mechanism as the 20S proteasome becomes the predominant proteasomal pathway in oxidative stress (Olshina et al., 2020; Deshmukh et al., 2023). This allows for a rapid, fine-tuned response in which damaged proteins are degraded but critical proteins are preserved as the redox-sensing mechanism facilitates fast termination of the oxidative stress response. Induction of this Nrf2 pathway in *Drosophila* increases proteasome subunit expression and decreases age-associated phenotypes (Tsakiri et al., 2013), while in human fibroblasts, this activation increases proteasome activity and delays cellular senescence (Kapeta et al., 2010). As cellular redox state normalizes to basal conditions, Nrf2 can be degraded by the 26S proteasome, terminating the oxidative stress response. This pathway is important in aging and neurodegenerative disease, and mutations in DJ-1 and NQO1 are associated with increased risk of developing PD and AD, respectively (Bian et al., 2008; Tsvetkov et al., 2011; Moscovitz et al., 2015).

### Protein aggregate formation in neurodegenerative diseases

Protein aggregates, a hallmark of neurodegenerative diseases, form through a complex interplay of factors that disrupt proteostasis including aging, chronic oxidative stress, mutations, breakdown of degradative pathways and chaperones, and errors during protein synthesis. Aggregation-prone proteins undergo structural changes in response to stress that increase disorder, form incorrect intramolecular bonds, and expose hydrophobic residues, making them targets for ubiquitin-independent degradation by the 20S proteasome (Kisselev et al., 2002; Saez and Vilchez, 2014; Nago et al., 2024). As aggregates form, they may sequester and deplete functional proteins, trigger inflammatory responses, and disrupt cellular membranes and signaling pathways, leading to a toxic cascade of neurodegenerative damage, cell death, and disease progression (Nago et al., 2024).

Aggregates are characteristic of neurodegenerative diseases, but they are not the most cytotoxic species of their constituent neurodegeneration-associated proteins, which include amyloid-β (Aβ) and tau in AD, mutant huntingtin (mHTT) in HD, and α-synuclein (α-syn) in PD. Instead, decades of data have indicated that the most damage is caused by misfolded soluble oligomers that interrupt cellular functions including proteasome degradation pathways (Takahashi et al., 2008; Tai et al., 2012; Usenovic et al., 2015; Fiolek T. et al., 2021). While early studies posited that oligomers inhibit proteasome activity by directly blocking the 20S pore or by acting as competitive substrates (Gregori et al., 1997; Zhao and Yang, 2010), more recent data show that at least three of these disease-associated oligomers (Aβ, α-syn, and HTT) act as allosteric inhibitors, forming a common three-dimensional conformation that allows them to bind and disrupt the 20S proteasome via stabilization of its closed state, thus preventing opening of its substrate entry gate and blocking access to HbYX motif-containing PAs and regulators like the 19S cap, PA200, and PI31 (Thibaudeau et al., 2018). This interaction further reduces the proteasome’s ability to degrade misfolded and aggregated proteins. As these oligomers accumulate and interact, formation of the large, insoluble aggregates may actually be a protective mechanism mitigating the effects of the toxic oligomers (Arrasate et al., 2004; Carrell et al., 2008; Boulos et al., 2024), although this is debated. Finally, the oligomers may interact with other regulators of the proteasome, impairing their activity (Olshina et al., 2020; Deshmukh et al., 2023).

### Therapeutic strategies to target ubiquitin-independent proteasome activity

Development of therapies for neurodegenerative disease have focused on enhancing clearance mechanisms, reducing and preventing misfolding, and eliminating toxic oligomers and aggregates. Most studies targeting the 20S proteasome for the treatment of neurodegenerative diseases have demonstrated that increasing 20S activation can reduce toxic protein aggregate levels *in vitro*, in cell culture, and in animal models of neurodegeneration (Pickhardt et al., 2005; Zhou et al., 2019; Cekala et al., 2022; Staerz et al., 2022; Sadahiro et al., 2024; Staerz et al., 2024), although at least one study has demonstrated that increases in certain PAs can increase neurodegenerative pathology (VerPlank et al., 2024). Activators that have been tested include activated endogenous PAs (as described in prior sections), peptidomimetics of PAs (Cekala et al., 2022; Cekala et al., 2024), as well as small molecule activators of 20S, such as fluspirilene analogs and dihydroquinazolines, which have been shown to rescue impaired proteasome activity and prevent pathological IDP aggregation of Aβ and α-syn (Fiolek T. et al., 2021; Fiolek T. J. et al., 2021). Drawbacks to using small molecule activators or activated endogenous PAs include a lack of specificity and the possibility of off-target effects in other essential cellular functions. Previously, there were no methods for targeting specific proteins to ubiquitin-independent proteasome pathways as there are for the UPS - called Proteolysis Target Chimeras (PROTAC) and molecular glues (Hyun and Shin, 2021) – meaning many activators could have off-target effects on essential proteins and cause more neuronal damage (Gao et al., 2020). However, several labs have recently published techniques, including chemical inducers of degradation (CIDEs) and direct-to-proteasome degraders (DPDs), to bypass the requirement for substrate polyubiquitination using chimera molecules or chemical dimerizers that directly target the desired substrate to the proteasome (Wilmington and Matouschek, 2016; Bashore et al., 2023; Balzarini et al., 2024). It is important to note that the value of 20S activators as disease-modifying therapies might also vary among different neurodegenerative diseases or at different stages of disease progression. Further research, especially *in vivo*, is necessary. Comprehensive reviews of recent therapeutic research on ubiquitin-independent proteasome mechanisms in age-related neurodegenerative disease can be found elsewhere (Harding and Tong, 2018; Opoku-Nsiah and Gestwicki, 2018; Hyun and Shin, 2021; Schmidt et al., 2021; Rawat et al., 2022; Tundo et al., 2023).

## Neurodegenerative disease-specific changes in ubiquitin-independent proteasome degradation

See Figure 2 and Table 1.

### Alzheimer’s disease

Alzheimer’s Disease (AD) is a progressive neurodegenerative disorder characterized by cognitive decline, memory loss, and neuropathological changes including aggregation of extracellular amyloid-β (Aβ) plaques and intracellular tau neurofibrillary tangles. While there is still debate about the mechanism by which each of these proteins contributes to neuronal deterioration, recent research has emphasized the role of synergistic crosstalk between the two proteins in producing AD pathology (Busche et al., 2019; Rawat et al., 2022; Roda et al., 2022). As in other age-related neurodegenerative diseases, oxidative stress, neuroinflammation, and disrupted proteostasis play important roles in the etiology of AD. Misfolding and posttranslational modifications induced by oxidation and inflammation interrupt the physiological functions of Aβ and tau in cytoskeletal support, recovery from injury, stabilization of microtubules, and synaptic plasticity (Haass and Selkoe, 2007; Orre et al., 2013; Bonet-Costa et al., 2016; Brothers et al., 2018), and this misfolding can cause toxic gain-of-function effects including disruption of normal protein degradation (Poppek et al., 2006; Opoku-Nsiah and Gestwicki, 2018; Davidson and Pickering, 2023), leading to cytotoxicity and cell death.

In AD, proteasome dysfunction is more severe than the decline associated with normal aging (Bonet-Costa et al., 2016). This compromised clearance pathway contributes to the formation of toxic protein oligomers that accumulate as aggregates. According to several studies, both tau and Aβ are IDPs that can be degraded by the proteasome through ubiquitin-independent mechanisms during physiological conditions, although alternative complementary degradative pathways may participate in degradation depending on cellular context or if the proteasome is inhibited (Grune et al., 2010; Zhao and Yang, 2010; Watanabe et al., 2020). In fact, based on mouse model data, impaired proteasome activity may induce AD pathology in individuals with an underlying diathesis. Prior to the development of pathology in AD model mice (3xTg-AD), impaired or inhibited proteasome activity can increase tau and Aβ accumulation, a process which can be rescued with Aβ immunotherapy against Aβ oligomers, reducing protein accumulation and restoring proteasome activity (Oddo et al., 2004; Oh et al., 2005; Tseng et al., 2008). Notably, proteasome activity can be inhibited during oxidative stress and neuroinflammation by CCRs, and mutations of CCRs that regulate cellular defense against oxidative stress, including NQO1, are associated with increased risk of developing AD (Bian et al., 2008; Tsvetkov et al., 2011; Moscovitz et al., 2015).

A study by Poppek et al. showed that tau is more resistant to oxidative stress than many other proteins, and oxidized tau is degraded equally as well as native, non-oxidized tau through the 20S proteasome *in vitro*. However, tau degradation in cells showed a different pattern. It was enhanced in cellular models (Poppek et al., 2006) of acute oxidative stress in which tau was oxidized and not phosphorylated but was strongly inhibited in models of chronic inflammation-induced oxidative stress, which caused hyper phosphorylation of tau, making it resistant to 20S proteasomal degradation. These data suggest an indirect mechanism affecting ubiquitin-independent proteasomal degradation wherein oxidized tau is rapidly degraded, but as the cell activates response pathways in chronic inflammation, tau is phosphorylated and the resulting hyperphosphorylated forms resist proteasomal degradation. Both ubiquitin-independent mechanisms and the UPS are impaired in AD, and hyperphosphorylated tau forms paired helical filaments that can directly bind to 20S proteasome complexes, affecting both 26S and 20S degradation mechanisms, and can associate into large neurofibrillary tangles (Keck et al., 2003; Poppek et al., 2006; Rankin et al., 2007; Min et al., 2010; Rawat et al., 2022).

Tau contributes to UPS dysfunction (Poppek et al., 2006; Tai et al., 2012; Thibaudeau et al., 2018), with higher levels of oligomeric and aggregated tau associated with a decrease in 26S activity without a decrease in subunit expression (Myeku et al., 2016). As discussed in the “*Protein Aggregation*” and “*Oxidative Stress*” sections of this review, soluble oligomers are the most toxic species of both tau and Aβ (Usenovic et al., 2015; Thibaudeau et al., 2018). Mouse models of AD show a physical association between tau and the 26S molecule that impairs degradation of ubiquitinated substrates and small peptides by the 26S and results in an increase in ubiquitinated protein burden. Treatment of healthy mice with tau oligomers also show decreasing degradative capacity, supporting the hypothesis that tau is proteotoxic, and this effect was rescued with activation of cAMP-protein kinase A (Myeku et al., 2016). It is worth noting, however, that the authors did not detect 20S activity, which has been shown in other studies to be active in AD brains (Gillardon et al., 2007; Türker et al., 2023) and to be directly inhibited by paired helical filament binding (Keck et al., 2003), so there may be underlying differences in experimental approaches that are important to revisit.

The other protein capable of forming aggregates characteristic of AD is Aβ, which has a direct inhibitory effect on proteasomal proteolytic pathways through allosteric stabilization of the 20S core particle in its closed state (Thibaudeau et al., 2018). Aged mouse models of AD overexpressing Aβ show a decrease in proteasome function that correlates with Aβ level, an effect which was reproduced in cultured neurons through extracellular Aβ application on cells (Favit et al., 2000; Oh et al., 2005) and *in vitro* (Gregori et al., 1995). Extracellular application of Aβ was specifically shown to affect chymotrypsin-like activity without affecting ubiquitination or deubiquitination levels, implying that the changes occurring in proteasomal pathways are due to direct effects on the proteasome rather than other UPS components (Gregori et al., 1995). Proteasome impairment correlates with Aβ oligomer levels but not aggregate levels (Tseng et al., 2008). Notably, although these and many other studies on Aβ and proteasomes are meant to describe the UPS, many do not distinguish between ubiquitin-dependent and ubiquitin-independent proteasome activity, so it is likely that at least some of the effects on proteasome activity are attributable to ubiquitin-independent mechanisms, especially as cellular stress increases (Zheng et al., 2016; Davidson and Pickering, 2023) and induces protein misfolding and the formation of toxic protein species.

In a comparative study of the inhibitory effects of Aβ monomers, oligomers, or fibrils on 20S activity, it was demonstrated that oligomers inhibit proteasomal degradation more than monomers or fibrils, supporting evidence that it is toxic oligomers rather than macroscopic aggregates that induce proteasome dysfunction (Tseng et al., 2008; Zhao and Yang, 2010). However, in another study, the opposite was found, with oligomeric and fibrillar Aβ increasing proteasome activity, and fibrillar species showing a much greater effect. This difference is likely due to a difference in experimental methods, with many groups using AMC peptide hydrolysis assays to measure activity and others using orthogonal approaches like activity ELISAs and activity-based probes (Orre et al., 2013; Türker et al., 2023).

Increasing data have shown that tau and Aβ each amplify the pathology of the other, driving an accelerated cycle of cellular dysfunction, resistance to and inhibition of proteasomal degradation, and neuronal deterioration. Aβ can trigger neuroinflammation and induce hyperphosphorylation of tau (Amadoro et al., 2011; Ising et al., 2019; Roda et al., 2022), paired helical filament formation, and tau aggregation. Conversely, excess misfolded tau causes Aβ aggregation, abnormal trafficking of the precursor protein for Aβ (APP), and increased prion-like propagation of both Aβ and tau pathology to other cells via exosomes and extracellular secretion of excess protein (Frost et al., 2009; Amadoro et al., 2011; Guo and Lee, 2011). As AD progresses, propelled by the damaging bidirectional pathway between tau and Aβ, changes occur to the composition of the proteasome, including significant upregulation of immunoproteasome subunits (Orre et al., 2013), and normal proteasomal degradation pathways are altered.

### Huntington’s disease

Huntington’s Disease (HD) is a progressive, autosomal dominant neurodegenerative disorder that causes motor dysfunction, mental health symptoms, cognitive decline, and eventually death. It is caused by a mutation in the N-terminus of the huntingtin (HTT) gene that leads to a large chain (>35–40) of glutamine amino acids, called a polyglutamine (polyQ) expansion, which is prone to misfolding, aggregation, and the formation of toxic peptide fragments and interferes with normal HTT roles in cellular trafficking, endocytosis, and transcription regulation, among others (Miller et al., 2011; El-Daher et al., 2015; Saudou and Humbert, 2016; Guo et al., 2018). Misfolded aggregates of mutant huntingtin (mHTT) form inclusion bodies, which are proposed to have both protective effects in reducing toxic peptide fragments and oligomers (Arrasate et al., 2004; Takahashi et al., 2008) as well as harmful effects through pathophysiological interactions and disruption of cellular processes, based on disease progression and cellular context (Waelter et al., 2001; Arrasate et al., 2004; Takahashi et al., 2008; Saudou and Humbert, 2016; Riguet et al., 2021).

The degradation pathways used for HTT breakdown likely vary by the species of HTT protein present (e.g., fragments, monomers, oligomers, aggregates). There is clear involvement of both autophagy and the UPS in HD, the roles of which are reviewed elsewhere (Martin et al., 2015; Sap et al., 2023), and the third degradative mechanism relevant to HD is ubiquitin-independent proteasomal degradation. mHTT has significant IDRs, especially in its polyQ region, which could make it a good substrate for the 20S proteasome and non-ATPase PAs (Juenemann et al., 2013). One *in vitro* study found that mammalian 20S proteasomes do not completely degrade polyQ repeats (Venkatraman et al., 2004), but many others have found the opposite, showing that the 20S can degrade wildtype HTT and mHTT (Rousseau et al., 2009; Juenemann et al., 2013) and that this effect is modulated with the addition of PA28αβ (Geijtenbeek et al., 2022). The 20S and ubiquitin-independent PAs may be especially critical in breaking down toxic mHTT fragments (Juenemann et al., 2013; Geijtenbeek et al., 2022), particularly as HD advances and the UPS is overwhelmed or compromised (Geijtenbeek et al., 2022). Indirectly, the 20S proteasome may also play a role in HD by maintaining overall proteostasis and mitigating the damage caused by oxidative and proteotoxic stress in neurons (Pickering et al., 2010; Höhn et al., 2020). More research is needed to directly show the extent to which ubiquitin-independent 20S proteasome activity drives degradation of mHTT and what regulatory and targeting mechanisms may exist.

While it was previously thought that HTT aggregates impair the UPS and proteasomal degradation by sequestering complexes within mHTT inclusion bodies (Holmberg et al., 2004), it is more likely that the co-localization of proteasome complexes and inclusion bodies reflects dynamic, targeted recruitment of ubiquitin and catalytically-active proteasomes that can facilitate both ubiquitin-dependent and ubiquitin-independent degradation (Schipper-Krom et al., 2014; Juenemann et al., 2018). In fact, 20S, 26S, PA28, PA200, and 19S all colocalize with perinuclear inclusions (Waelter et al., 2001; Aladdin et al., 2020), supporting evidence that multiple proteasomal pathways are involved in HTT clearance mechanisms.

Another component of the interaction between HD and ubiquitin-independent proteasomal degradation is the disruption of proteostasis pathways including the UPS. This could either increase ubiquitin-independent activity through the 20S or accompany disruption in ubiquitin-independent degradation depending on which proteasome molecules or regulators are most affected by the pathology of HD. Some studies do show an increase in proteasome activity in HD in early disease and in *postmortem* brains, likely providing a compensatory mechanism to account for proteostatic deficits elsewhere (Diaz-Hernandez et al., 2003; Thompson et al., 2009). Other studies demonstrate no deficits in 20S activity in proportion to protein aggregation, suggesting that aggregates do not directly impair proteasome activity (Diaz-Hernandez et al., 2003). Compatible with this research, data has suggested indirect mechanisms through which oligomers and aggregates impair proteasome activity, including mitochondrial dysfunction and disruption of overall proteostasis (Jana et al., 2001; Hipp et al., 2012). Another study using mHTT species derived from cells suggests that instead of aggregates, it is mHTT filaments, modified by posttranslational modifications, that impair proteasome function, and that these disruptions are especially harmful to 26S proteasomes rather than 20S, favoring a shift to 20S activity (Diaz-Hernandez et al., 2006). It is important to note that many *in vitro* studies use synthesized polyQ tracts, which do not have physiological posttranslational modifications and could diverge from native conditions. There is significant heterogeneity in results and conclusions drawn from the available literature regarding the effects of mHTT in its various forms on proteasome activity, and differences in experimental design are likely to explain some of the discrepancies.

Investigating interactions between mHTT and proteasomes can grant additional mechanistic insight. Some HTT found in HD cells is ubiquitinated, and data shows that ubiquitinated mHTT does not directly clog the 20S catalytic chamber in aggregate or soluble form (Hipp et al., 2012). However, when the concentration of mutant fragments reaches a certain threshold, cytoplasmic inclusions accumulate and deficits are observed in both the UPS and ubiquitin-independent mechanisms, likely demonstrating that it is an overall deficit in proteostasis rather than a dose-dependent effect of impaired ubiquitin conjugation mediating dysfunction (Hipp et al., 2012). Importantly, the authors note that in cells, most mHTT they found was not ubiquitin-conjugated, and that degradation of these fragments was not fully captured in their study.

There are various forms of ubiquitin-independent proteasomal degradation mechanisms which could have relevance in HD. One mechanism which has emerged is through alternative proteasome activator, PA28. *In vitro* studies have shown that a PA28γ mutant increases 20S catalytic activity and can promote complete degradation of polyQ peptides, suggesting that PAs may promote significant degradation of polyQ tracts synergistically with the 20S (Pratt and Rechsteiner, 2008). Wildtype PA28γ overexpression improved cell survival in excitotoxic and proteasome-inhibited states in a neuronal model of HD (Seo et al., 2007), and lentiviral-delivered gene therapy increasing PA28γ expression improved motor coordination in mouse models of HD and reduced ubiquitin-positive inclusion body expression, although the decrease in mHTT in inclusion bodies was not significant (Jeon et al., 2016). However, in a separate HD mouse model, it was noted that knockdown of PA28γ did not worsen polyQ-related pathology (Bett et al., 2006), so the exact effects of this PA in HD are not yet fully understood. In many cases, it is unclear what proportion of these effects is due to PA28γ interaction with the proteasome through ubiquitin-independent mechanisms *versus* its chaperone-like function or crosstalk with the UPS, and this will be an important area of future mechanistic study (Yersak et al., 2017). For example, in another polyQ-expansion disease, spinal and bulbar muscular atrophy, PA28γ had two opposing effects, increasing cell viability in association with its proteasome binding activity and conversely increasing aggregate formation and oligomer toxicity independently of its proteasome binding activity (Yersak et al., 2017). The effect of PA28γ is likely dependent on its cellular context.

In a study by Geijtenbeek et al., reduction in PAαβ activation in HD-model mice (R6/2) increased mHTT aggregation in the brain, and as the disease progressed, PA28αβ increasingly dissociated from the 20S proteasomes. This disassembly was specific to brain areas particularly affected by HD. This study also noted that *in vitro,* PA28αβ can enhance polyQ degradation through 20S proteasomes, but decreases overall mHTT degradation, implying that the regulatory effect of PA28αβ may be indirect (Geijtenbeek et al., 2022). Another recent study also found that PA28αβ can increase polyQ breakdown and suggested hybrid proteasomes may have a role (Kriachkov et al., 2023).

Another proteasome regulator and PA with relevance to HD is PA200, which is mainly found in the nucleus and recognizes short peptides and unstructured protein regions. Aladdin et al. (2020) recently demonstrated that human PA200 can bind to mHTT fragments and that the loss of PA200 in human cells contributed to aggregate formation and increased cytotoxicity. Additionally, the yeast ortholog of PA200 increased 20S degradation of soluble mHTT fragments *in vitro*, identifying that PA200-bound proteasomes may contribute to mHTT degradation, particularly in the nucleus (Aladdin et al., 2020). PA200 may also form hybrid proteasomes (Schmidt et al., 2005; Blickwedehl et al., 2008) and function in parallel to UPS-mediated degradation of ubiquitinated HTT to enhance digestion of disordered proteins, including aberrant species of HTT like soluble non-ubiquitinated polyQ sequences and oligomers.

In addition to the effects of PAs on proteasome activity, it is possible that HD pathology can induce an effect on proteasome composition. Diaz-Hernandez et al. found that the catalytic activity of the 20S core was preserved in HD mouse model brain extracts, and there was induction of immunoproteasome subunits LMP2 and LMP7 in the cortex and striatum of human brains and mouse models, with immunohistochemistry showing the highest expression in neurons. This induction only occurred after the development of significant HD pathology, and it was accompanied by reactive gliosis, with LMP2 and LMP7 also induced in nearby glia (Diaz-Hernandez et al., 2003). A follow-up study showed that expression of mHTT alone was insufficient to induce the observed changes in 20S activity but that mHTT works synergistically with IFNγ, causing an increase in immunoproteasome proportional to the severity of neuroinflammation-associated neurodegeneration (Diaz-Hernandez et al., 2004). Because immunoproteasomes can be beneficial in oxidative stress responses (Pickering et al., 2010), they likely serve a protective effect against damage from disrupted proteolysis and mitochondrial dysfunction.

There are many debates surrounding HD etiology, progression, and proteostasis. One possible contributor to these questions is that many studies in the literature presumed to describe the UPS ignore or do not control for ubiquitin-independent activity, and so it is possible that some of the complexity and contradictions in the literature about proteasomal pathways in HD are due to these diverging mechanisms. Further complicating the study of many neurodegenerative diseases, including HD, are the variations and limitations of model systems in reproducing critical aspects of the diseases (Bett et al., 2006; Seo et al., 2007; Ortega and Lucas, 2014). Additionally, some mechanisms may behave differently between *in vitro* experiments and cellular models, even within the same study (Geijtenbeek et al., 2022). The development of additional tools to study HD in physiological models will grant a better understanding of the biology of HD and inform the design of future studies and possible treatment avenues. Because the UPS becomes dysfunctional in late-stage HD (Jana et al., 2001), increasing degradation of mHTT by ubiquitin-independent 20S mechanisms could have therapeutic benefit. A recent review discusses therapeutic strategies in targeting different proteostasis pathways in HD (Harding and Tong, 2018).

### Parkinson’s disease

Parkinson’s Disease (PD) is a progressive, age-associated movement disorder caused by neurodegenerative changes primarily in dopaminergic neurons of the substantia nigra region of the brain. It is characterized by protein inclusions predominantly composed of aggregated α-synuclein (α-syn), called Lewy bodies, as well as failures of proteostasis, neuroinflammation, and oxidative and mitochondrial damage. In its physiological role, neuronal α-syn regulates neurotransmitter release through interaction with pre-synaptic membranes and synaptic vesicle release machinery (Calabresi et al., 2023). Different forms of α-syn are degraded through multiple proteasomal and lysosomal pathways, and impairment of one or more of these pathways can contribute to development of PD pathology and affect the proteolytic activity of other pathways (Ancolio et al., 2000; Webb et al., 2003; Arawaka et al., 2017; Stefanis et al., 2019).

In studying which pathways are used to degrade α-syn in normal and pathophysiological states, evidence has been mixed, and results conflict across experimental models and conditions, creating a complicated picture of intersecting proteolytic pathways, substrate degradation regulation through posttranslational modifications, and brain area-specific variations and vulnerabilities to neurodegenerative damage (Vogiatzi et al., 2008; Stefanis et al., 2019). For these reasons, data must be carefully compared across *in vitro*, cellular, and *in vivo* models when considering the broader PD proteostasis field. However, evidence in normal and PD-affected brains has consistently supported a role for ubiquitin-independent 20S proteasome degradation of α-syn, an intrinsically disordered protein (IDP) and known target of the 20S, especially in the context of oxidative stress (Tofaris et al., 2001; Machiya et al., 2010; Höhn et al., 2020; Coskuner-Weber et al., 2022). Like in other neurodegenerative diseases, mitochondrial dysfunction and oxidative stress are significant features of PD (Calabresi et al., 2023), and ubiquitin-independent proteasomal degradation is critical to clear accumulated damaged and oxidized proteins (Tofaris et al., 2001; Opoku-Nsiah and Gestwicki, 2018).

In PD, oxidative stress induces posttranslational modifications of α-syn including oxidation of methionines, which affect its degradation by the proteasome. Because cleavage of α-syn by the 20S requires first that the α-syn N-terminus binds to the 20S α7 subunit C-terminus, oxidation of α-syn N-terminal methionines during oxidative stress directly inhibits this degradation, slowing down clearance of α-syn and allowing accumulation within the cell (Alvarez-Castelao et al., 2014). Oxidized α-syn then continues to aggregate (forming non-fibrillar oligomers, then protofibrils, and finally fibrillar aggregates), becoming insoluble, and its degradation is significantly impaired relative to non-oxidized α-syn (Alvarez-Castelao et al., 2014). Certain modifications to α-syn that occur during oxidative stress are irreversible through normal pathways (Binolfi et al., 2016), and some of these modifications both inhibit protective modifications and facilitate harmful ones like phosphorylation of α-syn Serine-129 (pS129) (Anderson et al., 2006; Schildknecht et al., 2013). pS129 is an especially pathological modification present in over 90% of the α-syn in Lewy bodies, but under 4% of α-syn found in normal brains (Fujiwara et al., 2002; Anderson et al., 2006). Data in rat primary cortical cultures and SH-SY5Y neuroblastoma cells demonstrate that ubiquitin-independent proteasomal degradation is the primary mechanism through which pS129 α-syn in soluble monomeric form is degraded (Machiya et al., 2010), emphasizing the mechanism’s importance in mitigating pathology in PD. In insoluble form, pS129 α-syn is degraded both by ubiquitin-independent proteasome mechanisms and by the lysosome. However, after extensive aggregation, pS129 α-syn can no longer be degraded by the proteasome and collects within Lewy bodies (Arawaka et al., 2017). To relieve intracellular protein overload, α-syn can be exported into the extracellular space through exosomes, which can transfer α-syn between cells and may contribute to the spread of toxic α-syn species from cell-to-cell, nucleating pathological aggregation in those cells (Danzer et al., 2012; Lee et al., 2014; Stefanis et al., 2019). Extracellular α-syn can also induce neuroinflammation through activation of microglia, further compounding neurodegenerative changes in cellular stress (Lee et al., 2014; Calabresi et al., 2023).

As described in the “Catalytic Core Regulators” (CCRs) and “Oxidative Stress” sections, allosteric regulation of the 20S by CCRs is especially important during oxidative stress, and failure of the pathways regulating the oxidative stress response is central to PD pathology. Mutations in *PARK7*, the gene encoding PD-associated protein deglycase DJ-1 (also called Parkinson disease protein 7) increase vulnerability of cells to oxidative damage due in part to defects in regulation of ubiquitin-independent 20S proteasome degradation by DJ-1, which is upregulated in response to oxidative stress, coordinates the critically important Nrf2 antioxidant response pathway (see “*Oxidative Stress*” section), and allosterically inhibits ubiquitin-independent degradation by the 20S, protecting important physiological IDPs from degradation while allowing rapid destruction of damaged and oxidized proteins (Clements et al., 2006; Moscovitz et al., 2015; Deshmukh et al., 2023). The oxidation state of DJ-1 also affects the propensity of α-syn to form fibrils and affects its chaperone-like activity (Zhou et al., 2006).

Beyond changes to α-syn, as PD progresses, there are also alterations to proteasome expression and composition. These changes are most pronounced in areas most affected by PD pathology, like the substantia nigra pars compacta. McNaught et al. showed that in brains affected by sporadic PD, there are deficits in proteostasis associated with selective loss of 20S ⍺-subunits in dopaminergic neurons of the substantia nigra pars compacta but not in other areas (McNaught et al., 2002). Α-subunits, specifically α4, interact directly with parkin, an E3 ligase and one of the most important components of Parkinson’s disease.

The loss of these subunits causes structural instability of the proteasome and can prevent its coordinated assembly, contributing to the breakdown in proteostasis observed in PD and possibly the accumulation of Lewy bodies and dopaminergic cell death (McNaught et al., 2002). In addition, all three types of proteolytic activity in the 20S (chymotrypsin-like, trypsin-like, and caspase-like) are impaired in the substantia nigra of brains from patients who died of sporadic PD by up to 42% (McNaught and Jenner, 2001). Other studies have found that in neuronal cell models, the substantia nigra pars compacta may have lower baseline expression of PAs including 19S and PA28 and that dopaminergic neurons may not effectively upregulate PAs in response to stress as well as other cell types, making them increasingly vulnerable to damage by defective proteostasis (McNaught et al., 2010).

Mutations in parkin, an E3 ligase, can induce proteasome dysfunction. Parkin activates 26S in a ubiquitin-ligase-independent manner and enhances interactions between 19S subunits, playing a role in proteasome assembly that is disrupted by parkin mutations (Um et al., 2010). It also interacts directly with the α4 subunit of the 20S (Dachsel et al., 2005) and is proposed to have some function in substrate identification by the 20S/ubiquitin-independent pathways (Sanchez-Lanzas and Castano, 2014). A review by Sanchez-Lanzas and Castano (2014) describes several other 20S proteasome interactors and their relationships to ubiquitin-independent pathways.

## Conclusion

The proteasome is a cylindrical degradation complex composed of four stacked heptameric rings of α/β subunits (α_7_β_7_β_7_α_7_) around a central proteolytic chamber. Proteasomes catalyze the majority of protein degradation in mammalian cells through multiple mechanisms, the best characterized of which is the ubiquitin-proteasome system (UPS), a pathway employing ubiquitin protein tags, a 19S cap, and ATP hydrolysis to recognize, unfold, and degrade protein targets (Coux et al., 1996; Ciechanover, 1998; Ciechanover and Schwartz, 1998; Ben-Nissan and Sharon, 2014). Ubiquitin-independent proteasomal degradation is more recently studied and can be mediated by the 20S proteasome core or by a variety of proteasome activator complexes (e.g., PA28, PA200, and 19S) which facilitate degradation of unfolded and intrinsically disordered proteins without ATP (Opoku-Nsiah and Gestwicki, 2018). Increasing evidence supports a central role for ubiquitin-independent proteasome degradation in the oxidative stress response and clearance of protein aggregates in age-related neurodegenerative diseases (Kazee and Han, 1995; Lopez Salon et al., 2000; Bence et al., 2001; Iwata et al., 2005; Bennett et al., 2007; Wilson et al., 2011; Hipp et al., 2012; Ben Yehuda et al., 2017).

Literature on aging, neurodegenerative disease, and ubiquitin-independent 20S proteasome activity in different cellular contexts is ongoing and has had some conflicting results. Conflicts in the literature around the ubiquitin-independent proteasome are highly dependent on model system (species, *in vitro* vs. cultures vs. *in vivo*, etc.) and experimental design differences, and orthogonal approaches are necessary to definitively determine the behavior of the physiological system (Oh et al., 2005; Türker et al., 2023). In fact, it is possible some research describing the UPS in neurodegenerative disease reflects effects of both the UPS and ubiquitin-independent degradation because many studies rely on measures of global proteasome activity and pan-proteasome inhibitors, without isolating the ubiquitin-independent proteasome activity for assessment separately from the UPS. An additional barrier is the multifactorial changes associated with aging that complicate mechanistic investigations *in vivo*. As tools differentiating between proteasomal mechanisms are developed, the respective contributions of each mechanism to neurodegenerative disease may be better clarified.

Looking forward, many questions remain about how alternative mechanisms of protein degradation impact and are impacted by neurodegenerative processes. These include identifying how degradative pathways cooperate, determining how changes in proteasome activity and composition vary by cell type and cell compartment specificity in different diseases, further investigating the roles of alternative proteasome activator and regulators, investigating regulation of ubiquitin-independent proteasomal mechanisms by posttranslational modifications and interaction with binding partners, and confirming how these changes occur *in vivo*. Additionally, there have been recent developments in diverse neurodegenerative disease and proteasome research topics including liquid-liquid phase separation (Myers et al., 2018; Cohen-Kaplan et al., 2020; Yasuda et al., 2020; Zbinden et al., 2020; Hayashi et al., 2021; Mee Hayes et al., 2022; Hurtle et al., 2023), diagnostic and molecular tools (Devitt et al., 2018; Barthel et al., 2022), posttranslational 20S proteasomal processing of substrates for unique functions (Moorthy et al., 2006; Solomon et al., 2017), endoproteolytic proteasomal cleavage of disordered residues (Liu et al., 2003), proteasome-catalyzed peptide splicing (Liepe et al., 2016; Soh et al., 2024), ATP-independent 26S proteasomal degradation (Tsvetkov et al., 2020), which may further propel and nuance the understanding of proteasomal roles in neurodegenerative disease. Several of these topics are controversial and need more validation, but they reflect an appreciation of proteasome roles and regulation beyond the canonical UPS. Overall, this review examines the regulators, 20S-associated proteasome activators, and complexes involved in ubiquitin-independent proteasomal degradation, focusing on their bidirectional impact on age-associated neurodegenerative diseases.
